# Genetic Diversity, Natural Selection and Haplotype Grouping of *Plasmodium knowlesi* Gamma Protein Region II (PkγRII): Comparison with the Duffy Binding Protein (PkDBPαRII)

**DOI:** 10.1371/journal.pone.0155627

**Published:** 2016-05-19

**Authors:** Mun Yik Fong, Sarah A. A. Rashdi, Ruhani Yusof, Yee Ling Lau

**Affiliations:** Department of Parasitology, Faculty of Medicine, University of Malaya, Kuala Lumpur, Malaysia; Instituto de Higiene e Medicina Tropical, PORTUGAL

## Abstract

**Background:**

*Plasmodium knowlesi* is a simian malaria parasite that has been reported to cause malaria in humans in Southeast Asia. This parasite invades the erythrocytes of humans and of its natural host, the macaque *Macaca fascicularis*, via interaction between the Duffy binding protein region II (PkDBPαRII) and the Duffy antigen receptor on the host erythrocytes. In contrast, the *P*. *knowlesi* gamma protein region II (PkγRII) is not involved in the invasion of *P*. *knowlesi* into humans. PkγRII, however, mediates the invasion of *P*. *knowlesi* into the erythrocytes of *M*. *mulata*, a non-natural host of *P*. *knowlesi* via a hitherto unknown receptor. The haplotypes of PkDBPαRII in *P*. *knowlesi* isolates from Peninsular Malaysia and North Borneo have been shown to be genetically distinct and geographically clustered. Also, the PkDBPαRII was observed to be undergoing purifying (negative) selection. The present study aimed to determine whether similar phenomena occur in PkγRII.

**Methods:**

Blood samples from 78 knowlesi malaria patients were used. Forty-eight of the samples were from Peninsular Malaysia, and 30 were from Malaysia Borneo. The genomic DNA of the samples was extracted and used as template for the PCR amplification of the PkγRII. The PCR product was cloned and sequenced. The sequences obtained were analysed for genetic diversity and natural selection using MEGA6 and DnaSP (version 5.10.00) programmes. Genetic differentiation between the PkγRII of Peninsular Malaysia and North Borneo isolates was estimated using the Wright’s F_ST_ fixation index in DnaSP (version 5.10.00). Haplotype analysis was carried out using the Median-Joining approach in NETWORK (version 4.6.1.3).

**Results:**

A total of 78 PkγRII sequences was obtained. Comparative analysis showed that the PkγRII have similar range of haplotype (Hd) and nucleotide diversity (π) with that of PkDBPαRII. Other similarities between PkγRII and PkDBPαRII include undergoing purifying (negative) selection, geographical clustering of haplotypes, and high inter-population genetic differentiation (F_ST_ index). The main differences between PkγRII and PkDBPαRII include length polymorphism and no departure from neutrality (as measured by Tajima’s D statistics) in the PkγRII.

**Conclusion:**

Despite the biological difference between PkγRII and PkDBPαRII, both generally have similar genetic diversity level, natural selection, geographical haplotype clustering and inter-population genetic differentiation index.

## Introduction

Malaria is caused by protozoa of the genus *Plasmodium*. Four species are responsible for human malaria: *P*. *falciparum*, *P*. *vivax*, *P*. *malariae* and *P*. *ovale*. Human infections by the simian malaria parasite *P*. *knowlesi* have once been thought to be rare [1]. This was because *P*. *knowlesi* had been erroneously identified under microscopy examination as *P*. *malariae*, since both species have similar trophozoite morphology in the infected erythrocytes. This problem was eventually overcome by the use of highly specific polymerase chain reaction (PCR). Hence, a study at a district hospital in Sarawak (Malaysia Borneo) in 2004 reported PCR detection of a large number of knowlesi malaria cases that were initially diagnosed by microscopy examination as *P*. *malariae* infections [2]. Subsequent PCR tests on archived blood smears have also detected significant number of *P*. *knowlesi* infections. Human knowlesi malaria has now been documented in almost all countries in Southeast Asia [3].

*P*. *knowlesi* invades the erythrocytes of human and of its natural host, the macaque *Macaca fascicularis*, via interaction with the Duffy antigen on the erythrocytes [4–6]. The Duffy binding protein of *P*. *knowlesi* (PkDBP) is a large protein which is divided into seven regions (I-VII). Region II contains the critical motifs for binding to the erythrocyte Duffy antigen [5]. PkDBP is encoded by the α-gene, and is closely related to two other homologous proteins of *P*. *knowlesi*–Pkβ and Pkγ. However, unlike region II of PkDBPα (PkDBPαRII), the region II of β (PkβRII) and γ (PkγRII) does not binds to the Duffy antigen of human, but binds to rhesus monkey (*M*. *mulata*) erythrocytes [7]. Hence, Pkβ and Pkγ are responsible for the Duffy-independent pathways for invasion of rhesus erythrocytes. PkβRII binds to a sialic acid receptor on rhesus erythrocytes [8]. Although the receptor for PkγRII remains to be identified, it has recently been demonstrated that PkγRII is also a sialic acid-dependent invasion ligand [9].

Although PkDBPα and Pkγ are closely related erythrocyte binding proteins (EBP), each uses a different pathway to mediate invasion of *P*. *knowlesi* merozoite into the host erythrocyte. The PkDBPαRII binds with the Duffy protein receptor for chemokines (DARC) on the surface of erythrocytes, whereas PkγRII binds to N-glycolylneuraminicacid-sialylated receptors [9]. Previous studies [10, 11] have revealed high genetic diversity of PkDBPαRII in *P*. *knowlesi* isolates from Peninsular Malaysia and North Borneo. Also, the PkDBPαRII was observed to be undergoing purifying (negative) selection. Furthermore, phylogenetic analysis found that PkDBPαRII haplotypes of North Borneo isolates were genetically distinct from those of Peninsular Malaysia.

The aim of this study therefore is to determine whether PkγRII is subjected to the same selection forces as the PkDBPαRII, by measuring its genetic diversity level, selection trend (positive or negative) and geographical clustering of its haplotypes.

## Materials and Methods

### Ethics statement

Ethical clearance for this study was obtained from University of Malaya Medical Ethics Committee (Ref No. 817.18) and the Medical Research Ethic Committee (MREC), Ministry of Health, Malaysia (National Medical Research Register ID No.13079). Informed verbal consent was obtained from patients for use of their blood samples for diagnosis and research. Written consent was found to be unnecessary as verbal consent would be sufficient for the purpose of this study and patient details were noted down solely for personal recordkeeping. This consent procedure was approved by the ethics committees.

### Blood sample collection

Seventy-eight blood samples were used in this study. The samples (0.5 ml) were collected by trained medical personnel from knowlesi malaria patients in the University of Malaya Medical Centre (UMMC), Kuala Lumpur, Peninsular Malaysia (n = 48) and several public hospitals in two Malaysian states (Sabah and Sarawak) in North Borneo (n = 30). The samples were collected between 2010 and 2013.

### Extraction of DNA

Total DNA of *P*. *knowlesi* was extracted from each blood sample using the QIAGEN Blood DNA Extraction kit (QIAGEN, Hilden, Germany). In each extraction, 100 μl of blood was used. The extracted DNA was suspended in water to a final volume of 50 μl.

### PCR, cloning and sequencing of the PkγRII

The PkγRII was amplified by nested PCR using oligonucleotide primers F1: 5'-CGCATTTTGAAGGAATCCAC-3' and R1: 5'-TGCTAGACTTACCTTCACCT-3' for nest 1. The primers for the nest 2 reaction were F: 5’-TCCTCAAAAGGCGGTGACCATCC-3’ and R: 5’-ACTGGCTGCCTTAGATTCAACACCA-3’. Cycling conditions for nest 1 were as follows: 95°C for 4 mins, 30 cycles at 95°C for 30 secs, 48°C for 30 secs, and 72°C for 90 secs, followed by a 10 min extension at 72°C. The amplification for nest 2 was performed using the following cycling profile: 95°C for 4 mins, 30 cycles at 95°C for 30 secs, 56°C for 30 secs, and 72°C for 90 secs, followed by a 10 min extension at 72°C. The PCR products were purified with QIAquick Gel Extraction Kit and ligated into pGEM^®^-T plasmid vector (Promega Corp.,USA). Each ligation mixture was transformed into *Escherichia coli* TOP 10 competent cells. Plasmid DNA from clones having the desired DNA fragment was extracted using the QIAprep Spin Miniprep Kit. The plasmids were sent to a commercial laboratory (MyTACG Bioscience Enterprise, Malaysia) for DNA sequencing. To verify the sequences, the recombinant plasmids of three clones from each isolate were sequenced. In addition, the sequencing was performed in both strands of DNA.

### Sequence diversity, natural selection and haplotype analyses

Multiple alignment of PkγRII sequences was performed using CLUSTAL-Omega programme [12]. Both nucleotide and the deduced amino acid sequences were aligned and analysed. DnaSP ver. 5.10.00 [13] was used in the polymorphism analysis of the PkγRII sequences. Information such as haplotype diversity (Hd) and nucleotide diversity (π) was generated. The rates of synonymous (dS) and non-synonymous (dN) mutations were estimated and compared by the Z-test (P <0.05) in MEGA6 using the Nei and Gojobori’s method with Jukes and Cantor correction [14]. In purifying (negative) selection, mutations are usually not advantageous, so that dN will be less than dS (dN/*dS* < 1). However, in positive selection, non-synonymous mutations can be advantageous and dN will exceed dS (dN/dS > 1).

The Tajima’s D statistics [15] was used to test departures from the neutral theory of evolution, with the assumption that the population size was constant. The Wright’s F_ST_ fixation index [16] was used to measure genetic differentiation between the PkγRII of Peninsular Malaysia and North Borneo isolates. Both the Tajima’s D statistics and Wright’s F_ST_ index were determined using DnaSP 5.10.00. The Median-Joining method [17] in NETWORK v4.6.1.3 programme [18] was used to establish genetic relationship among the PkγRII haplotypes.

## Results and Discussion

The PkγRII of *P*. *knowlesi* clinical isolates from patients in Peninsular Malaysia and North Borneo were successfully amplified, cloned, and sequenced. Three clones from each isolate were used for sequencing. All clones from each isolate showed identical PkγRII sequences. A total of 78 PkγRII nucleotide sequences was obtained (GenBank Accession No. KR053974-KR054021, KU216673-KU216702). For analysis, the PkγRII sequence of the reference *P*. *knowlesi* strain H GenBank (GenBank Accession No. M90695) was also included. Each sequence was trimmed, using the codons for amino acids at the N- and C-terminals of PkDBPαRII as reference points.

Previous studies have found no length polymorphism in PkDBPαRII [10, 11]. All PkDBPαRII sequences of *P*. *knowlesi* isolates from Peninsular Malaysia and North Borneo were 307 amino acids in length. In the present study, multiple alignment of the nucleotide sequences revealed length polymorphism in the PkγRII gene, ranging between 915 and 921 base pairs, hence encoding amino acid sequences of 305–307 in length. Full alignment of these amino acid sequences (S1 Fig) showed 54 PkγRII sequences with 307 amino acids, and 24 sequences with 306 amino acids. The PkγRII of *P*. *knowlesi* strain H was the only sequence with 305 amino acids. Close inspection of the alignment showed two deletions in the PkγRII of *P*. *knowlesi* strain H, N at position 55 and K at position 166. For sequences with 306 amino acids, the deletion was K at position 166. Uniquely, no length polymorphism was seen in the North Borneo PkγRII sequences. All the North Borneo sequences were 307 amino acid in length.

The PkDBPαRII contains 12 conserved C residues (positions 16, 29, 36, 45, 99, 176, 214, 226, 231, 235, 304, 306) [11, 19]. These C residues form six disulphide bridges in the folding of PkDBPαRII for interaction with the erythrocyte Duffy antigen chemokine receptor (DARC). Surprisingly, despite having length polymorphism and no interaction with DARC, PkγRII still retained the 12 conserved C residues at same positions (S1 Fig) as in PkDBPαRII. Apart from the conserved C residues, the conserved residues Y94, N95, K96, R103, L168, and I175 are required for the interaction of PkDBPαRII with DARC [19]. In this regard, however, PkγRII showed no similarity with PkDBPαRII. Except for Y94, the remaining residues in PkγRII (S95, E96, K103, K168, N175) were different from those of PkDBPαRII. The amino acid changes at these key positions may possibly explain the inability of PkγRII to bind to human and *M*. *fascicularis* erythrocytes.

DNA sequence analyses were conducted to determine nucleotide diversity and genetic differentiation of PkγRII (Table 1). By taking the total 79 sequences as a single population, the overall haplotype diversity (Hd) and nucleotide diversity (π) were 0.991 ± 0.005 and 0.021 ± 0.001, respectively. To determine whether natural selection contributed to the diversity, the rate of non-synonymous (dN) to synonymous mutations (dS) was determined. dN (0.019) found to be lower than dS (0.030). The dN/dS ratio was 0.633, which suggested purifying (negative) selection of PkγRII. Although the overall Z-test did not show significant natural selection, there was indication towards purifying selection (dN < dS, *P* = 0.082). Tajima’s D statistics (-1.538, *P* > 0.10) revealed no significant departure from neutrality.

**Table 1:** Genetic diversity and selection pressure of PkγRII of *P*. *knowlesi* isolates in Peninsular Malaysia and North Borneo.

Origin	N	Hd ± SD	π ± SD	dN	dS	dN/dS	Z-test			Tajima’s D
							dN = dS	dN > dS	dN < dS	
Malaysia	79	0.991 ± 0.005	0.021 ± 0.001	0.019	0.030	0.633	*P* = 0.171^#^	*P* = 1.000^#^	*P* = 0.082^#^	-1.538^#^
Peninsular Malaysia	49	0.979 ± 0.013	0.009 ± 0.002	0.009	0.010	0.900	*P* = 0.706^#^	*P* = 1.000^#^	*P* = 0.375^#^	-2.352*
North Borneo	30	0.991 ± 0.011	0.013 ± 0.001	0.009	0.029	0.310	*P* = 0.004^&^	*P* = 1.000	*P* = 0.002*	-1.275^#^

N: number of sequences; Hd: haplotype diversity; π: nucleotide diversity; dN: rate of non-synonymous mutations; dS: rate of synonymous mutations

^#^ not significant (*P* > 0.10)

* significant (*P* < 0.01)

^&^ reject null hypothesis of strict neutrality, i.e., dN = dS

However, when the PkγRII sequences of Peninsular Malaysia and North Borneo were analysed as two separate populations, some differences were observed (Table 1). The North Borneo PkγRII was noted to have slightly higher diversity (Hd = 0.991 ± 0.011; π = 0.013 ± 0.001) than the PkγRII of Peninsular Malaysia (Hd = 0.979 ± 0.013; π = 0.009 ± 0.002). Tajima’s D statistics revealed significant departure of neutrality (-2.352, *P* < 0.01) only in the Peninsular Malaysia PkγRII. Although the dN/dS ratios showed purifying selection of PkγRII on both populations, the Z-test indicated strong purifying selection of the North Borneo PkγRII (dN < dS, *P* = 0.002). Comparative analysis showed that the PkDBPαRII of Peninsular Malaysia [9] and North Borneo [10] have almost similar range of haplotype (Hd = 0.986, 0.999) and nucleotide diversity (π = 0.013, 0.012) with that of PkγRII. Purifying selection on the PkDBPαRII was equal in both population (dN < dS, *P* < 0.05). Unlike PkγRII, the PkDBPαRII of both Peninsular Malaysia and North Borneo showed significant departure from neutrality by the Tajima’s D statistics (−2.085, *P* < 0.05; −2.459, *P* < 0.01 respectively) [9, 10].

It is interesting that higher haplotype diversity was seen in PkDBPαRII as compared to PkγRII the despite the lesser number of samples used in the PkDBPαRII studies [10, 11]. The previous and present studies used only human/clinical *P*. *knowlesi* isolates. Therefore, the PkDBαRII of the isolates is likely to be under selection pressure to generate high polymorphism (i.e., high haplotype diversity) for the parasite to escape the host's immune defenses. PkγRII, on the other hand, does not mediate invasion of *P*. *knowlesi* into human erythrocyte and therefore is likely under less selection pressure to display high polymorphism for immune evasion.

**Fig 1:**
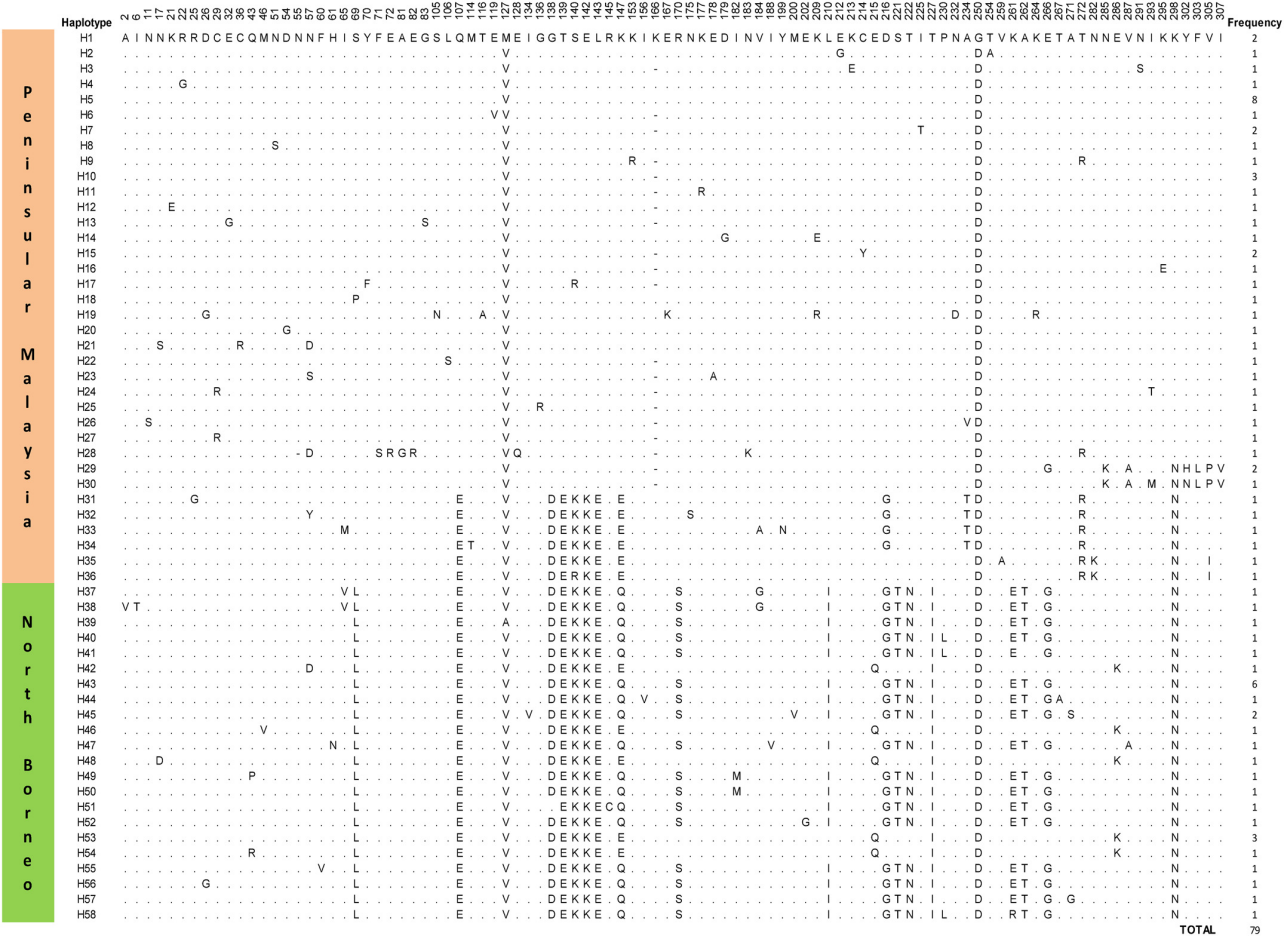
Amino acid sequence polymorphism in the PkγRII haplotypes from Peninsular Malaysia and North Borneo. Polymorphic amino acid residues are listed for each haplotype. Residues identical to those of haplotype H1 are marked by dots. Total number of sequences for each haplotype is listed in the panel on the right.

Phylogenetic analysis from a previous study reported two haplotype groups of PkDBPαRII in *P*. *knowlesi* from Peninsular Malaysia [10]. The distribution of the haplotypes though was uneven, because majority (83.3%) of the haplotypes clustered in a large or major group. A subsequent study reported distinct phylogenetic grouping of PkDBPαII haplotypes from North Borneo and Peninsular Malaysia [11]. In the present study, multiple alignment analysis identified 58 different PkγRII haplotypes (Fig 1). Thirty-six were from Peninsular Malaysia (H1-H36) and 22 from North Borneo (H37-H58). Haplotype H5 (frequency = 8/49) was the most abundant among the Peninsular Malaysia PkγRII haplotypes, while H43 (frequency = 6/30) was the most abundant in North Borneo. However, these frequencies were not significantly high to suggest that H5 and H43 were the most adapted haplotypes in the respective regions.

**Fig 2:**
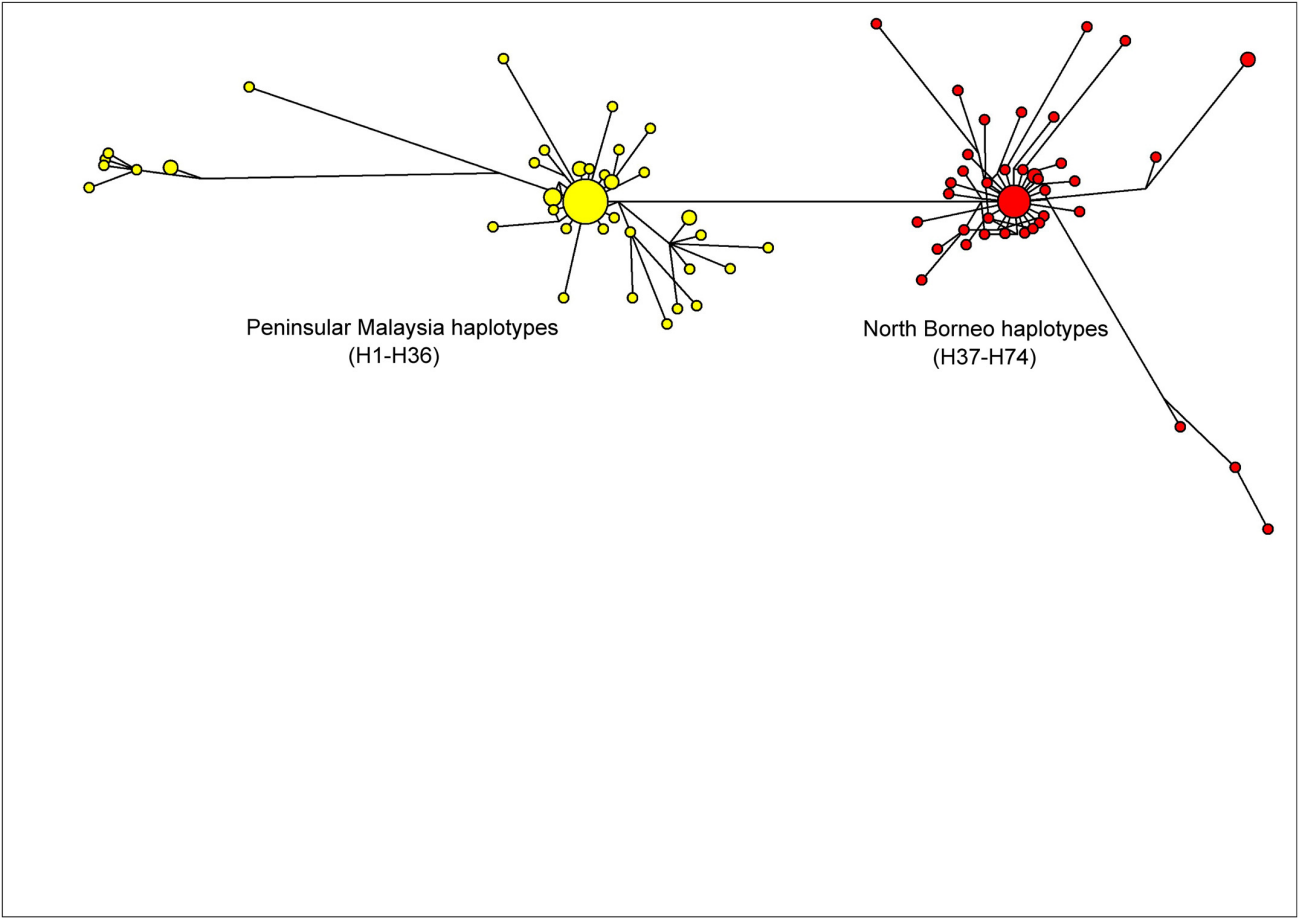
Median Joining network of PkDBPαRII haplotypes. The network shows geographical clustering of PkDBPαRII haplotypes from Peninsular Malaysia (yellow) and North Borneo (red). Amino acid sequences used for the construction of this network were from a previous study [9].

**Fig 3:**
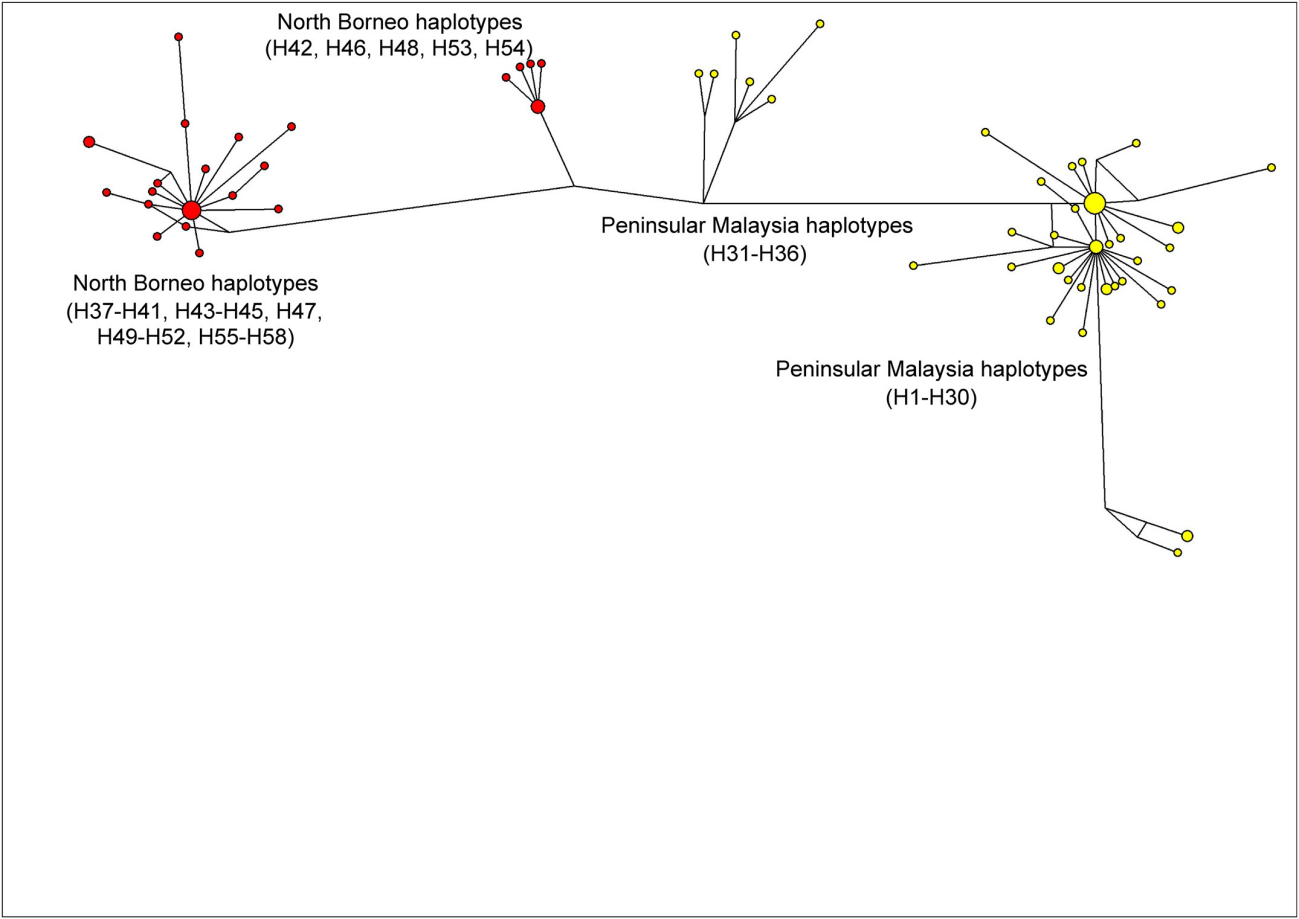
Median Joining network of PkγRII haplotypes. The network shows geographical clustering of PkγRII haplotypes from Peninsular Malaysia (yellow) and North Borneo (red). Note that haplotypes from each region are divided into two subgroups.

The previous studies on PkDBPαRII used Neighbour Joining to establish haplotype groupings and relationship. The present study, however, used the Median-Joining method [17] in order to obtain a more precise depiction of haplotype relationship or network. Similar to the Neighbour Joining approach reported previously [11], the Median-Joining method generated a PkDBPαRII haplotype network consisting of distinct Peninsular Malaysia and North Borneo groups (Fig 2). For PkγRII haplotypes, a more complex network was obtained (Fig 3). Like PkDBPαRII, geographical clustering of Peninsular Malaysia and North Borneo haplotypes was evident. However, haplotypes from each region were separated into two subgroups. The large sub-groups were comparatively more complex, with high number of haplotypes. Interestingly, the short link connecting the small subgroups (Peninsular Malaysia’s H31-H36, and North Borneo’s H42, H46, H48, H53, H54) suggests close relationship among the haplotypes. This was attributed to the sharing of some common amino acid residues in these haplotypes, namely, E107, D138, E139, K140, K142, E143 and E147. In contrast to PkDBPαRII and PkγRII, the highly diverse *P*. *knowlesi* circumsporozoite Th2R/Th3R epitope region displays no geographical clustering of haplotypes, and many shared haplotypes from Peninsular Malaysia and Malaysian Borneo were observed in the Median Joining Network [20].

The Wright’s F_ST_ fixation index measures population differentiation due to genetic structure [16]. The index is also a measure of gene flow between populations. Populations with F_ST_ values of more than > 0.25 are considered to be highly differentiated. A high Wright’s F_ST_ index (0.61) was reported between the PkDBPαII of Peninsular Malaysia and North Borneo, indicating significant genetic difference between the haplotypes groups [11]. The F_ST_ index obtained in this study was equally high (0.62), thus showing high genetic difference between the PkγRII of Peninsular Malaysia and North Borneo. The amino acid substitutions in the PkγRII which most likely contribute to this genetic difference were at positions 69, 107, 138, 139, 140, 142, 143, 170, 216, 221, 222, 227, 261, 262 and 266 (Fig 1). Similarly, a recent study based on microsatellite DNA data F_ST_ found high level of genetic differentiation between *P*. *knowlesi* human isolates from Peninsular Malaysia and Borneo [21]. The observation of high F_ST_ values between *P*. *knowlesi* populations from Peninsular Malaysia and Malaysian Borneo is likely due to the separation of these two land masses since the last ice age 65,000 years ago. The *P*. *knowlesi*, its macaque hosts and mosquito vectors in Borneo became isolated, and subsequently diverged from their respective species on mainland Asia to form distinct subpopulations. Molecular phylogeny and evolutionary studies have revealed unique history of macaque group formation in Borneo Island [22, 23]. The *Anopheles* mosquitoes have formed species which are uniquely found in the island, such *An*. *latens* and *An*. *balabacensis*, both of which are vectors of *P*. *knowlesi* [24, 25]. Therefore, the geographical divergence of *P*. *knowlesi* is most likely the result of host immune selection and adaptation in the macaque and vector hosts respectively.

The population substructure may also explain the Tajima’s D statistics observed in this study. The absence of a significant Tajima’s D < 0 in the total sample (-1.538, *P* > 0.10) could be linked to the high level of subdivision between the Peninsular and Borneo Malaysia population as revealed by the distribution of the haplotype network (Fig 3) and F_ST_ index. However, since Tajima’s D tends to be > 0 in the presence of subdivision, it may be the case that this tendency is cancelling out the signal of Tajima’s D negativity observed in the Malaysia Borneo population (-1.275, *P* > 0.10).

Although human knowlesi malaria is generally mild, there has been an increase in the number of severe infections accompanied by high parasitaemias [26]. There is evidence to suggest possible human-to-human transmission [27]. Furthermore, *P*. *knowlesi* has been observed to expand its preferred host cell niche by invading older red blood cells [28]. All this may suggest increased parasite adaptation to humans. Rapid genetic changes in the invasion ligands including PkγRII may play a role in this increased adaptation in humans.

## Conclusions

The region II of PkDBPα and Pkγ plays an important role in the invasion of *P*. *knowlesi* into host erythrocytes. PkDBPαRII binds to the Duffy antigen of human and *M*. *fascicularis* erythrocytes, but PkγRII binds to a hitherto unidentified receptor on *M*. *mulatta* erythrocytes. Despite this difference, PkγRII was found to be almost similar to PkDBPαRII with regards to genetic diversity level, natural selection, geographical haplotype clustering and genetic differentiation index.

## Supporting Information

S1 Fig Full amino acid sequence alignment of *Plasmodium knowlesi* gamma protein region II (PkγRII). Amino acid residues identical to those of *P*. *knowlesi* strain H (GenBank Accession No. M90695) are indicated by dots. Dash indicates amino acid deletion (highlighted blue at positions 55 and 166). The twelve conserved C residues are highlighted in yellow. The crucial amino acid residues in PkDBPαRII required for interaction with DARC are at Y94, N95, K96, R103, L168 and I175. In PkγRII, these positions have changed (highlighted orange at S95, E96, K103, K168, N175) except at Y94 (highlighted green).
(XLSX)
